# Heat-killed *Bifidobacterium longum* BBMN68 and inulin protect against high-fat diet-induced obesity by modulating gut microbiota

**DOI:** 10.3389/fnut.2024.1406070

**Published:** 2024-08-14

**Authors:** Siyuan Sun, Qi Zhang, Dongdong Li, Hongliang Li, Hairan Ma, Xiuying Wu, Yixuan Li, Pengjie Wang, Rong Liu, Haihong Feng, Yongxiang Zhang, Yue Sang, Bing Fang, Ran Wang

**Affiliations:** ^1^Key Laboratory of Functional Dairy, Department of Nutrition and Health, China Agricultural University, Beijing, China; ^2^Inner Mongolia Mengniu Dairy (Group) Co., Ltd., Hohhot, Inner Mongolia, China; ^3^Mengniu Hi-Tech Dairy (Beijing) Co., Ltd., Beijing, China; ^4^Research Center for Probiotics, China Agricultural University, Beijing, China; ^5^College of Food Science and Engineering, Gansu Agricultural University, Lanzhou, China

**Keywords:** *Bifidobacterium longum* BBMN68, inulin, obesity, gut microbiota, heat-killed

## Abstract

**Introduction:**

Obesity, a pervasive global epidemic, has heightened susceptibility to chronic ailments and diminished the overall life expectancy on a global scale. Probiotics and inulin (IN) have been documented to mitigate obesity by exerting an influence on the composition of the gut microbiota. Whether heat-killed *Bifidobacterium longum* BBMN68 (MN68) and IN have an anti-obesity effect remains to be investigated.

**Methods:**

In this study, Wistar rats were fed a high-fat diet (HFD), and orally administered heat-killed MN68 (2 × 10^11^ CFU/kg) and/or inulin (0.25 kg/kg) for 12 weeks. Histological analysis, serology analysis and 16S rRNA gene sequencing were performed.

**Results:**

Heat-killed MN68 + IN treatment showed an enhanced effect on preventing weight gain, diminishing fat accumulation, and regulating lipid metabolism, compared to either heat-killed MN68 treatment or inulin treatment. Gut microbiota results showed that heat-killed MN68 + IN treatment significantly increased the relative abundance of *Bacteroidota*, *Oscillospira*, *Intestinimonas*, *Christensenella*, and *Candidatus_Stoquefichus*, and reduced the relative abundance of *Enterococcus*. Furthermore, heat-killed MN68 + IN significantly increased the SCFA levels, which were correlated with changes in the gut microbiota.

**Discussion:**

This research provides support for the application of heat-killed MN68 and IN in the treatment of obesity, and highlights the combination of heat-killed BBMN68 and IN as functional food ingredients.

## 1 Introduction

The prevalence of obesity has become a serious health concern globally. Obesity, specifically excess lipid accumulation, substantially increases the risk of several diseases like type 2 diabetes, cardiovascular disease, and cancers (1). Fat reduction surgery, physical exercises, and drugs are commonly used to prevent obesity. However, all of these treatment have their inherent drawbacks: fat reduction surgeries are expensive and carry risks such as infection, bleeding, and regaining weight; physical exercises seems challengeable due to time constraints and lack of time effectiveness; drugs have several side effects, including drug dependence, abdominal pain, and abdominal distension (2). In recent decades, it has been established that gut microbiota dysbiosis is causally linked to the onset of obesity. Studies have shown that the gut microbiota participates in the decomposition and synthesis of fat through different mechanisms (3). Hence, there is an imminent requirement to discern safe and efficacious natural products for averting obesity through the modulation of gut microbiota.

Postbiotics are suitable candidates for preventing chronic disease, which is currently a very interesting topic (4). Compared with living bacteria, postbiotics possess no risk of bacteria translocation and are easy to standardize (5). Moreover, recent studies have shown that heat-killed or fragmented *lactobacillus* strains (such as CP1563, Lr263, and HK L-137) can ameliorate obesity-induced metabolic abnormalities or adipose tissue inflammation (6–8). Postbiotics contribute to obesity prevention through several mechanisms, including the enhancement of intestinal permeability, modulation of the gut microbiota and its metabolites (such as short-chain fatty acids, SCFAs) (8), and the regulation of hormone levels in the intestine to exert control over energy metabolism (9). Consequently, leveraging postbiotics stands as a promising strategy in the prevention of obesity.

Inulin is widely regarded as one of the most effective and frequently employed prebiotics for the modulation of gut microbiota (10). Studies have shown that inulin treatment leads to gut microbiota remodeling and the SCFAs increase in obese mice, which enhances the expression of angiopoietin-like protein 4 (ANGPTL4) and regulates lipid metabolism (11). Similar research revealed that inulin protects against obesity by nourishing gut microbiota to restore IL-22-mediated enterocyte function in mice going on a high-fat diet (HFD) (11, 12). In a placebo-controlled randomized trial, researchers found that oligofructose-enriched inulin selectively altered the gut microbiota and significantly reduced weight z score, body fat percentage, and serum IL-6 levels in overweight or obese children (13). It has also been shown that the incorporation of *Lactobacillus acidophilus* and inulin can improve lipid metabolism and biochemical parameters in mice with HFD (14). Nevertheless, the impact of combining inulin with heat-killed probiotics for the prevention of obesity remains unexplored.

*Bifidobacterium longum* BBMN68 (MN68) was isolated from the centenarian feces in Bama, China. It exerts beneficial effects like improving immunity and maintaining the integrity of the gut barrier (15). Previous studies have also explored the bile tolerance and adhesion mechanism of BBMN68, which indicates that MN68 could be used as probiotics for improving body immunity, reducing allergic responses, and enhancing the intestinal digestion function (16, 17). In this study, an HFD-induced obese rat model was established to determine if heat-killed MN68 and inulin could prevent obesity, and 16S rRNA gene sequencing method was used to analyze the gut microbiota. We aim to provide a theoretical basis for the combined use of heat-killed MN68 and inulin as natural food ingredients for preventing obesity.

## 2 Materials and methods

### 2.1 Animals and diets

Specific pathogen-free (SPF) male Wistar rats (4–5 weeks old) were obtained from Vital River Laboratory Animal Technology Co Ltd., Beijing, China. Sterilized water and standard rodent chow (Beijing University Health Science Center, Beijing, China) were provided *ad libitum* throughout the experiment. All procedures involving animals were conducted by the Guidelines in the Care and Use of Animals. The study was reviewed and approved by the Animal Studies Committee of the Health Science Center, Institute of Medicinal Plant Development, Chinese Academy of Medical Sciences, Beijing, China (Approval number: PONY-2021-FL-58).

**Figure 1:**
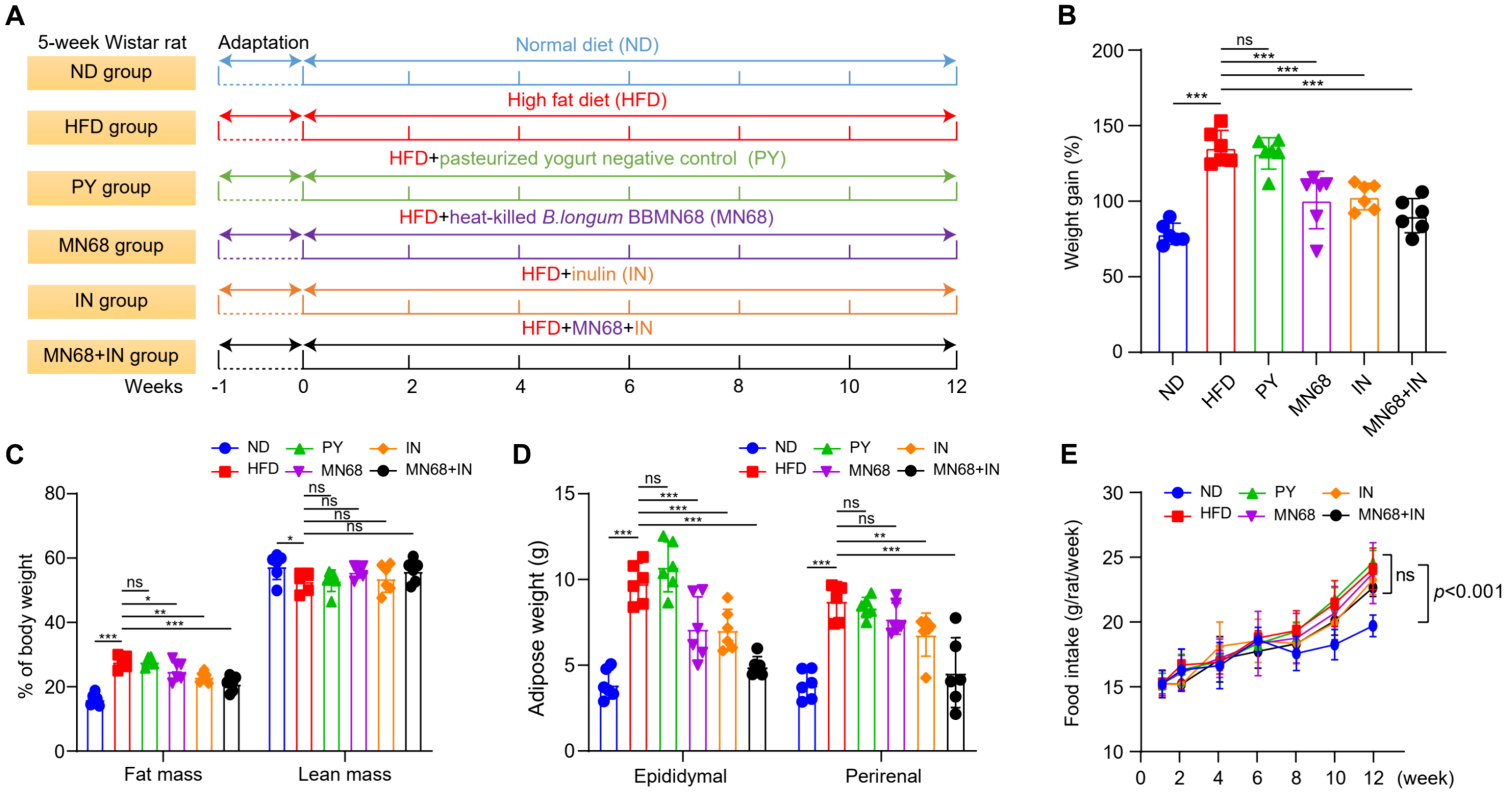
Heat-killed MN68 and inulin reduce body weight and fat accumulation in HFD-induced obesity rats. Experimental workflow in rats **(A)**. Body weight gain after 12 weeks **(B)**. Fat mass and lean mass percentage of body weight **(C)**. Total white fat weight from different organs **(D)**. Food intake **(E)**. Rats were divided into six groups (per group *n* = 6): ND, normal diet; HFD, high-fat diet; PY, pasteurized yogurt treatment; IN, pasteurized yogurt with inulin treatment; MN68, pasteurized yogurt with BBMN68 treatment; MN68 + IN, pasteurized yogurt with both inulin and BBMN68 treatment. Data were presented as the means (M) ± standard deviation (SD). *p* values were determined by a one-way ANOVA with LSD test. *p < 0.05; **p < 0.01, ***p < 0.001.

The rats were maintained in a temperature-controlled environment (22°C ± 2°C) with a light–dark cycle alternating between 12 h of light and 12 h of darkness. For establishing the obesity prevention model, 36 rats were equally divided into six groups. After acclimatizing the rats with the basal diet for 1 week in individual cages, the rats were fed test diets for 12 weeks. The control group (two groups of rats) received physiological saline, where one control group was fed the normal diet (3.85 gm%), and the second control group was fed the high-fat diet (4.73 gm%, Table 1). The remaining four groups fed on the fat-rich diet received pasteurized yogurt alone, inulin and heat-killed BBMN68 supplements alone, and a combination of inulin and heat-killed BBMN68 supplements, respectively. All components were administered via oral gavage (1 mL/rat) every day for 12 weeks, and the experimental outline was shown in Figure 1A. The body fat rate and lean meat percentage were measured by small animal magnetic resonance imaging (MRI) system-permanent magnet MRI NM21-060H-I (Suzhou Niumag Analytical Instrument Co., Ltd., China), and the fecal samples were collected before the rats were euthanized and stored at −80°C for further use.

**Table 1:** Composition of normal diet and high-fat diet for rats.

	High-fat diet (Batch No. SYHF45)		Normal diet (Batch No. SYC50H)	
	gm%	kcal%	gm%	kcal%
Protein	23.7	20	19.2	20
Carbohydrate	41.4	35	67.3	70
Fat	23.6	45	4.3	10
Kcal/gm	4.73		3.85	
Ingredient	gm	kcal	gm	kcal
Casein	200	800	200	800
L-Cystine	2	12	3	12
Corn Starch	72.8	291	452.2	1808.8
Maltodextrin	100	400	75	300
Sucrose	172.8	691	172.8	691
Cellulose	50	0	50	0
Soybean Oil	25	225	25	225
Lard	177.5	1,598	20	180
Mineral Mix S10026	10	0	10	0
DiCalcium Carbonate	13	0	13	0
Calcium Carbonate	5.5	0	5.5	0
Potassium Citrate, 1H_2_O	16.5	0	16.5	0
Vitamin Mix V10001	10	40	10	40
Choline Bitartrate	2	0	2	0
FD&C Yellow Dye #5	0	0	0.04	0
FD&C Red Dye #40	0	0	0.01	0
FD&C Blue Dye #1	0.05	0	0	0
Total	858.15	4,057	1055.05	4,057

The unit of “gm” is the short name of “gram”.

### 2.2 Preparation of the test samples

BBMN68 was cultured in De Man, Rogosa, and Sharpe (MRS) broth supplemented with 2% maltose for 24 h. After repeated culturing, BBMN68 was centrifuged at 5000 rpm for 10 min. The pellet was then dissolved in phosphate buffered solution (PBS), and BBMN68 was autoclaved at 121°C for 15 min. After BBMN68 was heat-killed and inactivated, they were powdered and mixed with pasteurized yogurt. The pasteurized yogurt was an excipient for probiotic samples. To prevent the influence of pasteurized yogurt, a pasteurized yogurt control (PY) group was also established. In the IN group, inulin supplements were added to make the final concentration of 0.25 g/mL and in the MN68 group, 2 × 10^8^ CFU/mL heat-inactivated BBMN68 was used. In the MN68 + IN group, the same concentrations of inulin and heat-killed BBMN68 were used.

### 2.3 Analysis of biochemical parameters in serum

After 12 weeks, blood samples were collected from the abdominal aorta after rats were fasted overnight, and then centrifuged at 1000 × *g* for 15 min to isolate the serum, which were stored at −80°C for further use. Lipid metabolism indexes in serum including fasting plasma glucose (FPG), fasting insulin (FINS), total cholesterol (TC), triglyceride (TG), high-density lipoprotein-cholesterol (HDL-C), and low-density lipoprotein-cholesterol (LDL-C) were determined by HITACHI Automatic Analyzer 3100. The concentrations of leptin, adiponectin, lipopolysaccharide (LPS), interleukin (IL)-10, IL-1β, IL-6, IL-4, IL-10, tumor necrosis factor-α (TNF-α), and interferon-γ (IFN-γ) were measured by using commercially available enzyme-linked immunosorbent assay (ELISA) kits [Multisciences (Lianke) Biotech, Co., Ltd., Hangzhou, China].

### 2.4 Histological analysis

The epididymal adipose tissues were fixed in 4% paraformaldehyde and embedded in paraffin. The paraffin-embedded sections were stained using hematoxylin and eosin (H&E). The histopathology of liver tissues and epididymal adipocyte was visualized using an optical microscope (Leica DM6 B). The number and the area of the adipocytes were calculated using ImageJ 1.52v software (Wayne Rasband, National Institutes of Health, USA).

### 2.5 SCFAs analysis

Acetate, propionate, and butyrate concentrations were measured as described previously (18). Briefly, 25 mg of fecal sample was mixed with 500 μL of purified water containing 0.5% phosphoric acid, and the samples were ground at freezing temperature. The samples were placed in centrifuge tubes and treated with ultrasonic sound for 10 min at an ultrasonic power of 1,000 watts, followed by centrifugation at 13,000 × *g* for 15 min. Next, 200 μL of n-butanol (10 μg/mL 2-ethylbutyric acid as the internal standard) was added to the 200 μL supernatant for extraction and vortexed for 10 s. The samples were then treated using ultrasonic sound at 4°C for 10 min and centrifuged at 13000 × *g* for 5 min. The supernatant was collected and filtered through a 0.22 μm membrane. The concentration of SCFAs was measured using a gas chromatographic-mass spectrometer 8890B-7000D (GC–MS, Agilent J&W Scientific, Folsom, CA, USA) equipped with the HP-FFAP column (30 m × 0.25 mm × 0.25 μm, Agilent Technologies, Inc., Santa Clara, CA, USA). The temperature of the splitless injector was 250°C, the injection volume was 1 μL, and the carrier gas used was nitrogen. The temperature program of the GC oven was set as follows: starting at 80°C for 5 min, increased by 20°C/min to 120°C for 2 min, increased by 5°C/min to 160°C for 8 min, and maintained at 220°C for 3 min. The temperature of the electron impact ion source was 230°C, and the electron energy was 70 eV. The MassHunter software v10.0.707.0 (Agilent, USA) was used to analyze and calculate the final concentrations of different SCFAs.

### 2.6 16S rRNA sequencing and gut microbiota analysis

DNA extracted from the fecal samples of mice was amplified, and 16S rDNA sequencing was performed. The primers were designed for the variable V3–V4 regions and a PCR instrument (ABI GeneAmp^®^ 9700 type) was performed to amplify DNA fragments of samples. Each experiment was carried out in triplicate. The amplified PCR products were mixed and resolved on 2% agarose gel. The AxyPrepDNA gel extraction kit (AXYGEN company) was used to extract PCR products, diluted with Tris–HCl, and the PCR products were quantified using QuantiFluor^™^ -ST blue fluorescence quantification system (Promega, Wisconsin, United States). The Miseq library was built using TruSeq^™^ DNA Sample Prep Kit (Illumina), and PCR products were sequenced using the Illumina platform. The sequencing data is accessible at the link http://www.ncbi.nlm.nih.gov/bioproject/911911 with BioProject ID: PRJNA911911.

For downstream analysis, quality control of paired-end raw sequencing reads was performed using Fastp (version 0.19.6). Merging of the reads was conducted using Flash (version 1.2.11) as follows: (1) Bases with quality scores below 20 were trimmed from the end of reads using a sliding window of 50 bp, discarding reads shorter than 50 bp and those containing ambiguous bases (N); (2) Paired-end reads were merged based on overlap, with a minimum overlap length of 10 bp; (3) The maximum allowable mismatch ratio in the overlap region was set to 0.2, with non-conforming sequences being filtered out; and (4) Sequences were demultiplexed and reoriented according to barcodes and primers, with no mismatches allowed in barcodes and up to two mismatches allowed in primers. Normalization was achieved by rarefying all sample sequences to the same reads for subsequent analyses. For taxonomic profiling, the RDP classifier^1^ was used to align sequences against the Silva 16S rRNA gene database (v138) with a confidence threshold of 70%. All reads were clustered into the operational taxonomic units (OTUs) using Usearch (version 7.1), which are defined by a 97% identity threshold of the 16S rRNA sequences. For the bioinformatics analysis, Majorbio Cloud (Majorbio Bio-Pharm Technology Co., Ltd., Shanghai, China), a web-based free platform, was used to perform the Wilcoxon rank-sum test and to construct Spearman Correlation Heatmap and the Linear discriminant analysis effect size (LEfSe) plot.

### 2.7 Statistical analysis

All data are represented as mean (M) ± standard deviation (SD). The data on body weight, fat accumulation, and serum levels were analyzed using one-way ANOVA with LSD test. SPSS statistics 26 was used for statistical analysis, and *p* < 0.05 was considered statistically significant.

## 3 Results

### 3.1 MN68 + IN treatment effectively reduces obesity-related parameters in obese rats

After 12 weeks of administering supplements via oral gavage, a significant reduction in the body weight of rats in the MN68 and IN group was observed compared to the HFD and IN group; the weight gain in rats from MN68, IN, and MN68 + IN group was all significantly less compared to the HFD group (*p* < 0.05) (Figures 1A,B). Regarding body composition, the body fat mass in the MN68 + IN group was significantly less (*p* < 0.05) than those in both the HFD and PY groups. No significant difference in the percentage of lean mass was found between the MN68 + IN group and the HFD group (Figure 1C). Significant reductions in both epididymal and perirenal adipose weight were observed in the IN and MN68 + IN groups compared to the HFD and PY groups (Figure 1D). There were no significant differences in the food intake among the HFD groups (Figure 1E). The rats in the IN and MN68 groups showed less obesity extent compared to the rats in the HFD group. Therefore, the combination of heat-killed MN68 and inulin showed a favorable delayed process of obesity induced by a high-fat diet.

### 3.2 MN68 + IN treatment changes liver and adipose histology and regulates the lipid metabolism indexes

**Figure 2:**
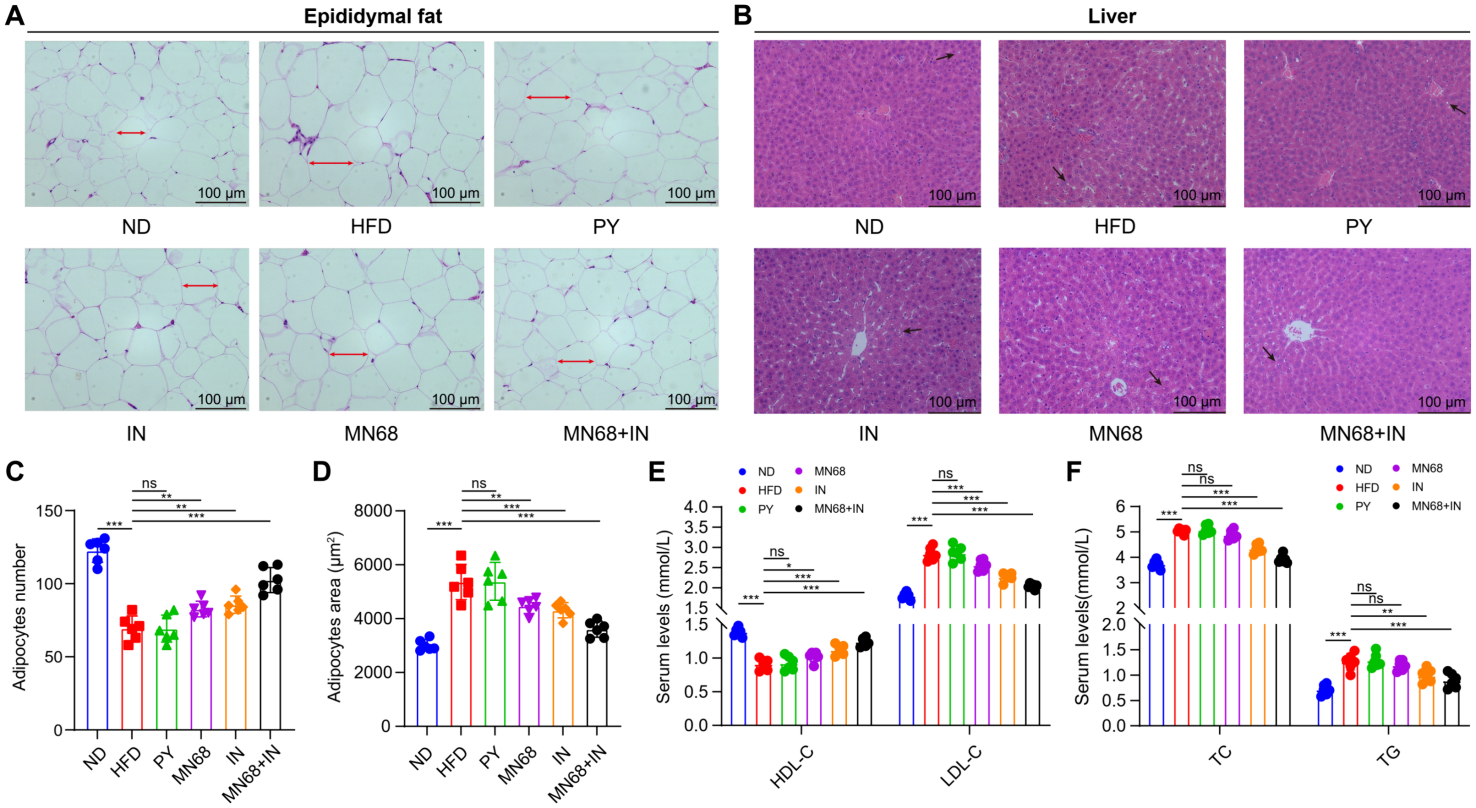
Heat-killed MN68 and inulin improve serum lipid metabolism indexes, and liver and adipose tissue morphology in HFD-induced obesity rats. H&E staining of epididymal fat tissue **(A)** and liver tissue **(B)**. Epididymal adipose cell measurement indexes include adipocyte number **(C)** and adipocyte area **(D)**. Lipid metabolism indexes in serum include TC (total cholesterol), TG (triglyceride), HDL-C (high-density lipoprotein-cholesterol), and LDL-C (low-density lipoprotein-cholesterol) **(E,F)**. Rats were divided into six groups (per group *n* = 6): ND, normal diet; HFD, high-fat diet; PY, pasteurized yogurt treatment; IN, pasteurized yogurt with inulin treatment; MN68, pasteurized yogurt with BBMN68 treatment; MN68 + IN, pasteurized yogurt with both inulin and BBMN68 treatment. Data were presented as the means (M) ± standard deviation (SD). *p* values were determined by a one-way ANOVA with LSD test. *p < 0.05; **p < 0.01, ***p < 0.001.

The histopathological changes in both epididymal adipose tissues and liver tissues were observed under the microscope (Figures 2A,B). The H&E staining morphological analysis revealed that the liver and adipose tissue cells in the ND group were normal and orderly. Compared to the ND group, the liver tissue of the HFD group showed slight hepatocyte degeneration and lipid droplet infiltration. The liver lesions of IN, MN68, and MN68 + IN groups were reduced, and the number of lipid vacuoles was reduced. The outline of adipose tissue cells in the HFD group was larger than that in the ND group, and the arrangement was looser. The volume of adipocytes in IN, MN68, and MN68 + IN groups was smaller and the cell arrangement was tighter compared with the HFD group. The size of the adipocytes was measured, and the results showed that the size of adipocytes in the MN68, IN, and MN68 + IN group was significantly less (*p* < 0.05) compared to the HFD group (Figures 2C,D).

Serum lipid metabolism indexes including TC, TG, HDL-C, and LDL-C were detected in the six experimental groups. Compared to the ND group, HFD treatment significantly increased the serum TC, TG, and LDL-C levels, and significantly decreased the HDL-C level (*p* < 0.05, Figures 2E,F). There were no obvious differences in the serum levels of the four lipid metabolism indexes among the HFD group and the PY group. After MN68 treatment for 12 weeks, the serum TC and LDL-C were significantly decreased (*p* < 0.05), and there was no significant change in the TG levels between the HFD and MN68 groups (*p* > 0.05). Compared to the HFD group, a significant decrease (*p* < 0.05) in TC, TG, and LDL-C concentrations was observed in the IN, and MN68 + IN groups, with the MN68 + IN group having the most significant effect. Thus, these results demonstrate that Heat-killed MN68 and inulin improve serum lipid metabolism indexes, and liver and adipose tissue morphology in HFD-induced obesity rats, and their combination has a better effect.

To analyze the influence of heat-killed MN68 and inulin on serum biochemical parameters in HFD-induced obesity rats, serum leptin, adiponectin, LPS, and inflammatory factors levels were detected among the six groups. As shown in Table 2, rats in the HFD group were significantly changed compared to the ND group. Heat-killed MN68 + IN had significantly effect on reducing the LPS, IL-1β, and IFN-γ levels, and lifting the IL-4 and IL-10 levels. Nevertheless, heat-killed MN68 and inulin had no significant effect on the serum leptin, adiponectin, IL-6, and TNF-α levels. Moreover, serum FPG, FINS, and HOMA-IR levels were compared to explore the effect of heat-killed MN68 and inulin on insulin resistance in HFD-induced obesity rats. Compared to the ND group, a significant increase in FPG and FINS concentrations were observed in the HFD group (*p* < 0.05). MN68 + IN treatment significantly decreased the FPG and HOMA-IR levels, but there was no significant difference in the FINS level compared to the HFD group.

**Table 2:** Heat-killed MN68 and inulin regulate serum biochemical parameters and insulin resistance in HFD-induced obesity rats.

Serum biochemical parameters	Groups					
	ND	HFD	PY	MN68	IN	MN68 + IN
Leptin (ng/mL)	1.996 ± 0.235^a^	2.077 ± 0.454^a^	2.176 ± 0.344^a^	1.992 ± 0.185^a^	2.101 ± 0.239^a^	2.270 ± 0.243^a^
Adiponectin (μg/mL)	14.304 ± 1.372^a^	11.415 ± 1.701^b^	11.883 ± 0.957^b^	12.478 ± 1.222^b^	12.589 ± 0.909^b^	12.119 ± 1.109^b^
LPS (EU/mL)	0.299 ± 0.038^d^	0.599 ± 0.060^a^	0.613 ± 0.058^a^	0.505 ± 0.043^b^	0.472 ± 0.047^bc^	0.407 ± 0.076^c^
IL-1β (pg/mL)	34.285 ± 2.216^d^	70.255 ± 5.749^a^	69.689 ± 4.735^a^	60.276 ± 6.600^b^	53.142 ± 6.976^c^	48.935 ± 7.352^c^
IL-6 (pg/mL)	18.905 ± 1.221^b^	26.077 ± 4.029^a^	25.658 ± 4.908^a^	25.200 ± 3.355^a^	28.823 ± 4.223^a^	26.572 ± 2.264^a^
TNF-α (pg/ml)	0.764 ± 0.078^b^	0.920 ± 0.069^a^	0.918 ± 0.140^a^	0.860 ± 0.102^ab^	0.827 ± 0.117^ab^	0.862 ± 0.098^ab^
IFN-γ (pg/mL)	19.657 ± 3.291^d^	40.160 ± 3.896^a^	38.669 ± 3.819^a^	29.485 ± 4.622^b^	26.844 ± 2.715^bc^	23.236 ± 2.008^cd^
IL-4 (pg/mL)	2.988 ± 0.282^a^	2.704 ± 0.224^c^	2.711 ± 0.235^c^	2.751 ± 0.227^c^	2.774 ± 0.223^c^	2.855 ± 0.309^b^
IL-10 (pg/mL)	7.712 ± 0.413^a^	4.963 ± 0.443^d^	4.809 ± 0.454^d^	5.059 ± 0.544^d^	6.011 ± 0.561^c^	6.866 ± 0.394^b^
FPG (mmol/L)	5.610 ± 0.084^c^	6.755 ± 0.117^a^	6.653 ± 0.102^ab^	6.687 ± 0.126^ab^	6.570 ± 0.184^b^	6.558 ± 0.113^b^
FINS (μU/mL)	2.845 ± 0.055^b^	3.062 ± 0.069^a^	3.055 ± 0.095^a^	3.043 ± 0.065^a^	3.008 ± 0.066^a^	3.010 ± 0.047^a^
HOMA-IR	0.710 ± 0.020^c^	0.922 ± 0.026^a^	0.903 ± 0.027^ab^	0.905 ± 0.034^ab^	0.878 ± 0.026^b^	0.877 ± 0.024^b^

Rats were divided into six groups (per group *n* = 6): ND, normal diet; HFD, high-fat diet; PY, pasteurized yogurt treatment; IN, pasteurized yogurt with inulin treatment; MN68, pasteurized yogurt with BBMN68 treatment; MN68 + IN, pasteurized yogurt with both inulin and BBMN68 treatment. Data were presented as the means (M) ± standard deviation (SD). *p* values were determined by a one-way ANOVA with LSD test, and different lowercase letters (e.g., a, b, c) indicate significant differences, *p* < 0.05. LPS, lipopolysaccharide; IL, interleukin; TNF-α, tumor necrosis factor-α; IFN-γ, interferon-γ; FPG, fasting plasma glucose; FINS, fasting insulin; HOMA-IR, homeostasis model assessment of insulin resistance; HOMA-IR was calculated by FPG (mmol/L) × FINS (μU/mL)/22.5.

### 3.3 MN68 + IN treatment regulates the gut microbiota composition

#### 3.3.1 Diversity of gut microbiota

**Figure 3:**
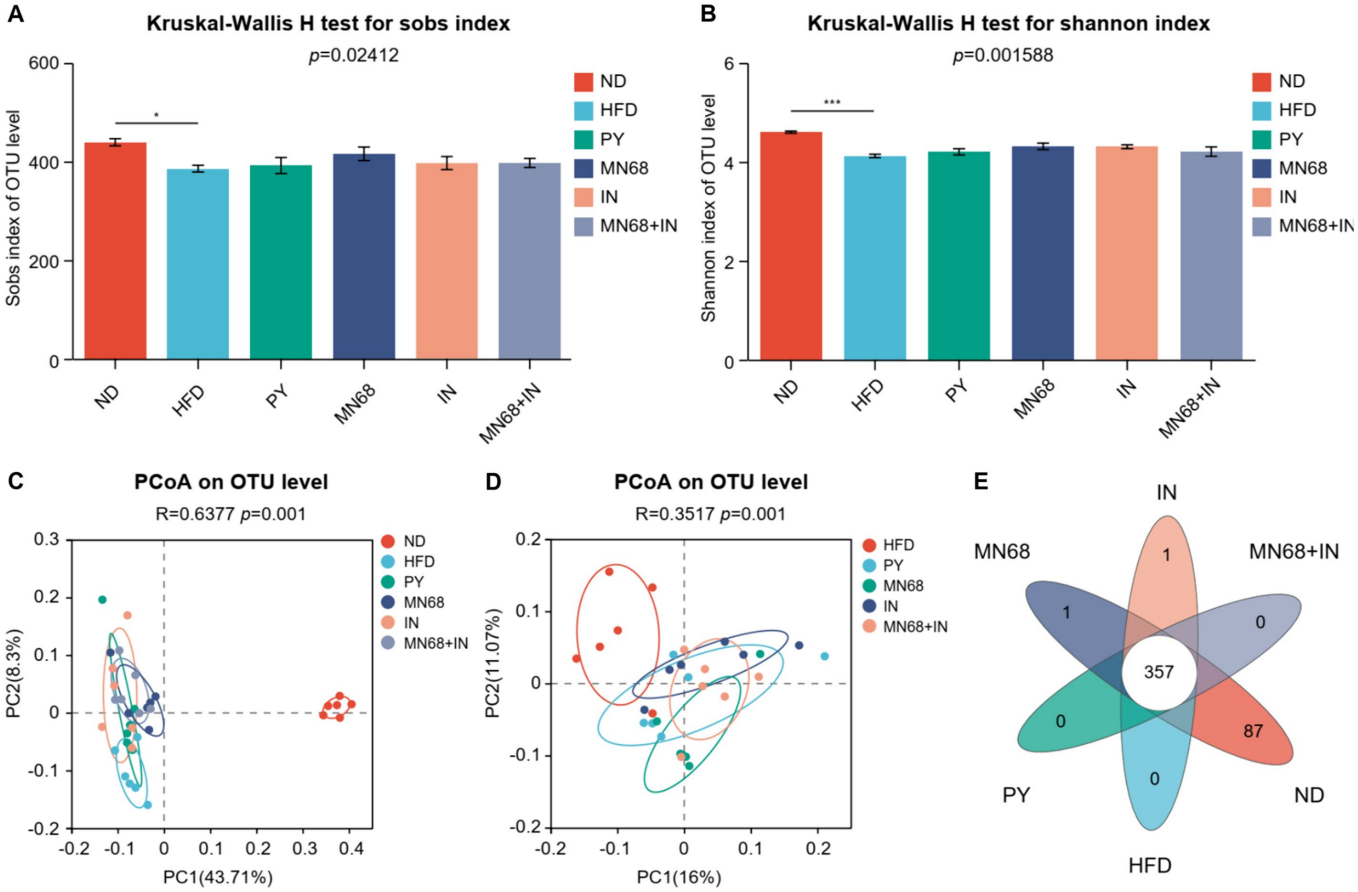
Heat-killed MN68 and inulin alter the gut microbiota diversity in HFD-induced obesity rats. Sobs **(A)** and Shannon **(B)** indexes of α-diversity. PCoA plot of β-diversity based on the OTU among groups **(C,D)**. Venn diagram with the overlapping area showing the shared OTUs among groups **(E)**. Rats were divided into six groups (per group *n* = 6): ND, normal diet; HFD, high-fat diet; PY, pasteurized yogurt treatment; IN, pasteurized yogurt with inulin treatment; MN68, pasteurized yogurt with BBMN68 treatment; MN68 + IN, pasteurized yogurt with both inulin and BBMN68 treatment. Data were presented as the means (M) ± standard deviation (SD). *p* values were determined by a one-way ANOVA with the Kruskal-Wallis test. *p < 0.05; **p < 0.01; ***p < 0.001.

16S rDNA sequencing was performed to explore the microbial diversity in the fecal samples of rats in different groups after 12 weeks of oral gavage. The abundance of α-diversity, including *Sobs*, and *Shannon* indexes of the microbiota of rats in the ND group, was significantly higher compared to the other groups (Figures 3A,B). However, no significant differences in the abundance of α-diversity were observed in other groups. Principal coordinate analysis revealed a significant difference (*p* = 0.001) in the composition of the gut microbiota in all groups (Figure 3C). The dots of the ND group were departed from other groups while the dots of the MN68 + IN groups were departed from the HFD group (Figure 3D). Venn diagrams show that the OTU number of the ND group was much more than other groups (Figure 3E).

#### 3.3.2 The composition of the microbial community at the phylum and genus level

**Figure 4:**
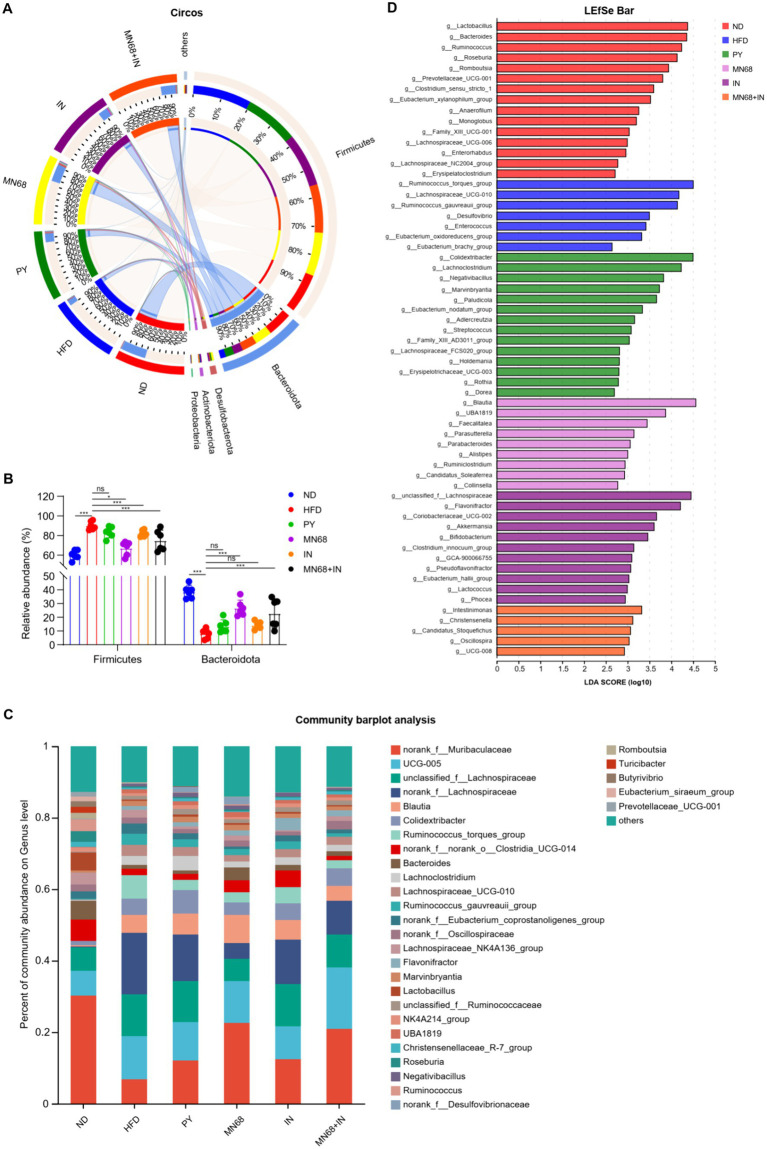
Heat-killed MN68 and inulin change the gut microbiota composition on phylum and genus levels in HFD-induced obesity rats. **(A)** Circos graph of the phyla microbiota among the six groups. **(B)** Relative abundance of *Bacteroidota* and *Firmicutes*. **(C)** Microbiota composition at the genus level. **(D)** *LEfSe* analysis among the six groups. Rats were divided into six groups (per group *n* = 6): ND, normal diet; HFD, high-fat diet; PY, pasteurized yogurt treatment; IN, pasteurized yogurt with inulin treatment; MN68, pasteurized yogurt with BBMN68 treatment; MN68 + IN, pasteurized yogurt with both inulin and BBMN68 treatment. Data were presented as the means (M) ± standard deviation (SD). *p* values were determined by a one-way ANOVA with Kruskal-Wallis test and Wilcoxon rank-sum test. *p < 0.05; **p < 0.01, ***p < 0.001.

As for the phyla microbiota, *Bacteroidota* and *Firmicutes* were the two main phyla among the six groups (Figures 4A,B). The abundance of *Bacteroidota* in the MN68 + IN group was significantly higher (*p* < 0.05) than those in the HFD and PY groups with the abundance of *Firmicutes* showing the opposite trend. Relative abundances at the genus level were shown in Figure 4C, and different abundances were found among the six groups. Excluding the genera categorized as “no-rank” and “unclassified,” the 10 genera exhibiting the highest relative abundance include *Blautia*, *Colidextribacter*, *Ruminococcus_torques_group*, *Bacteroides*, *Lachnoclostridium*, *Lachnospiraceae_UCG-010, Ruminococcus_gauvreauii_group*, *Lachnospiraceae_NK4A136_group*, *Flavonifractor*, *Marvinbryantia*, and *Lactobacillus* (Figure 4C). The *LEfSe* analysis showed that *Blautia*, and *Faecalitalea* were significantly enriched in the MN68 group, and *Akkermansia*, *Bifidobacterium*, and *Lactococcus* were enriched in the IN group (Figure 4D). As for the MN68 + IN group, the genera *Oscillospira*, *Intestinimonas, Christensenella,* and *Candidatus_Stoquefichus* were significantly enriched.

#### 3.3.3 MN68 + IN treatment modulates SCFA-related gut microbiota and increases SCFA production

**Figure 5:**
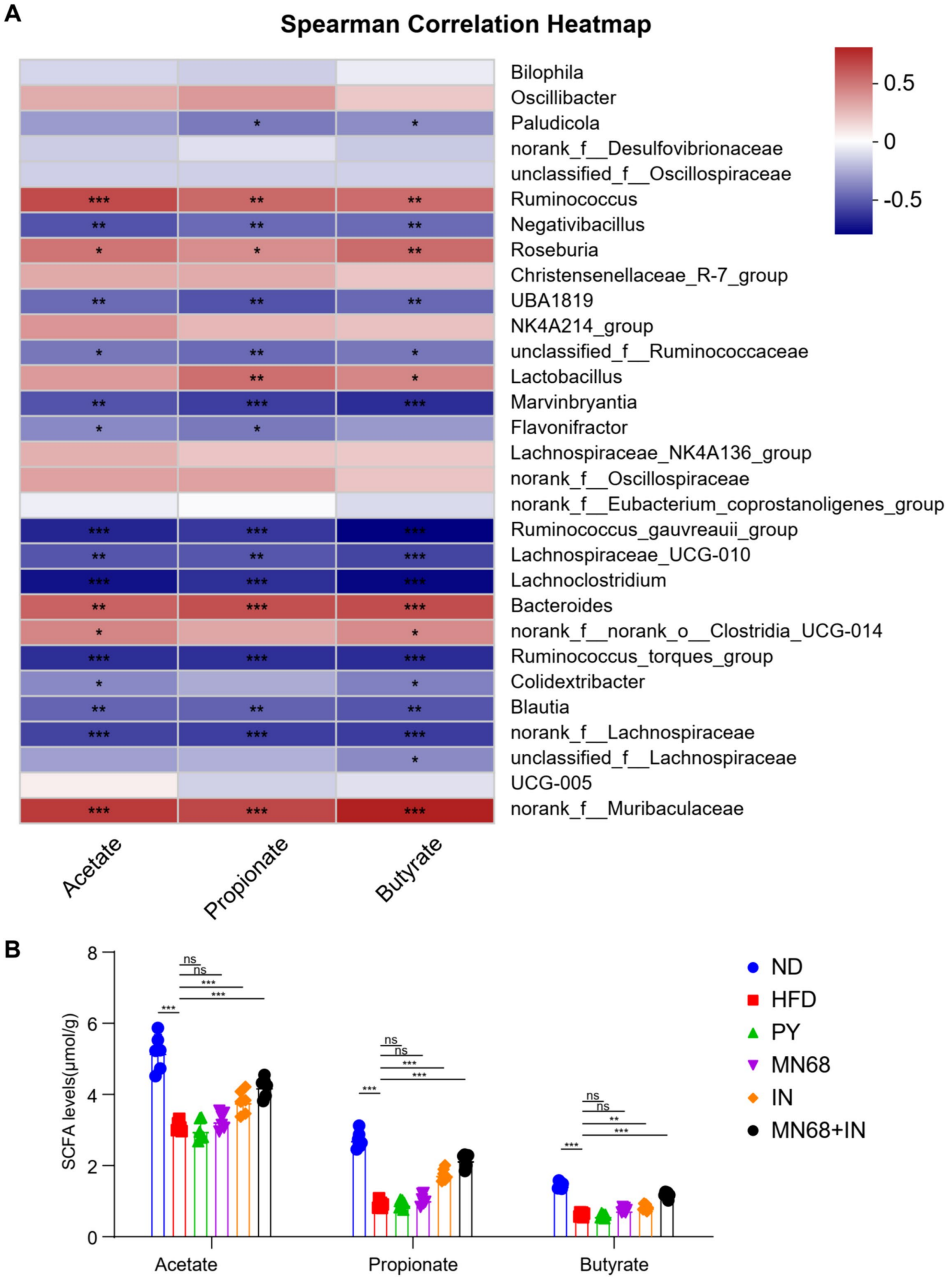
Heat-killed MN68 and inulin increase the SCFA level by regulating gut microbiota composition in HFD-induced obesity rats and regulating the relative expression of obesity-related genes. **(A)** Spearman correlation heatmap between gut microbiota and SCFA level. Red indicates a positive correlation coefficient, while blue indicates a negative correlation coefficient. **(B)** SCFA level among the six groups. Rats were divided into six groups (per group *n* = 6): ND, normal diet; HFD, high-fat diet; PY, pasteurized yogurt treatment; IN, pasteurized yogurt with inulin treatment; MN68, pasteurized yogurt with BBMN68 treatment; MN68 + IN, pasteurized yogurt with both inulin and BBMN68 treatment. Data were presented as the means (M) ± standard deviation (SD). *p* values were determined by a one-way ANOVA with LSD test and Wilcoxon rank-sum test. *p < 0.05; **p < 0.01, ***p < 0.001.

Spearman correlation analysis was performed to study the correlation of the SCFA and the distribution of genera in fecal microbiota in all mice, and the results are shown in Figure 5A. The genera *Ruminococcus, Roseburia, Lactobacillus,* and *Bacteroides* were found to have a positive correlation with all acetate, propionate, and butyrate concentration. The concentrations of acetate, propionate, and butyrate were measured (Figure 5B). The results showed a significant increase (*p* < 0.05) in the levels of acetate, propionate, and butyrate in rats in the MN68 + IN group compared to the rats supplemented heat-killed MN68 or inulin alone, which tended to recover the original concentrations of SCFAs in the gut of rats in the ND group.

## 4 Discussion

The prevalence of obesity is high globally, so there is an urgent need to explore new effective methods to prevent and treat obesity. Mounting evidence has demonstrated that gut microbiota plays a key role in obesity development (19), and novel therapeutic approaches, such as the use of probiotics, prebiotics, and postbiotics, could aid in preventing obesity (20). Previous studies have shown that *Bifidobacterium longum* BBMN68 improves intestinal functions and immunity (15, 21). However, the potential of heat-killed BBMN68 as a postbiotic is yet to be explored. Moreover, yogurt is redefined as a carrier for probiotic food and could benefit individuals who are obese by regulating their appetite and improving intestinal barrier function as well as lipid profiles (22). However, the function of pasteurized yogurt, an ideal matrix for delivering nonviable bacteria, is still unknown. Therefore, in this study, we have determined the effect of oral administration of pasteurized yogurt containing heat-killed BBMN68 and inulin on obesity prevention using the HFD-induced Wistar rat model.

For the successful establishment of the HFD-induced obesity model, the average weight of animals in the HFD group should be 20% higher compared to the ND group (23). In our study, the average weights of rats in the ND and HFD groups were 422.12 g and 568.68 g, respectively, thus indicating that the obesity model was successfully established. HFD increases lipids in the body, which are then deposited on the white adipose tissues like epididymal adipose tissue (18). We analyzed the weight gain in rats in different groups, and the results demonstrated that the weight gain in the rats in the MN68 + IN group was significantly less, and the rats were relatively slimmer compared to the HFD and PY groups. Furthermore, a significant reduction in weight gain, fat body rate, epididymal fat weight, and adipocyte size was observed in the rats in the MN68 + IN group compared to the IN group and the MN68 group. This indicates that MN68 + IN could effectively alleviate and prevent obesity. Together, these results show that heat-killed BBMN68 and inulin have an enhanced effect on preventing obesity.

Leptin and adiponectin are the key adipokines secreted by the adipocytes. Leptin resistance is characterized by high levels of leptin in serum, typically observed in patients with nutritional obesity (24). Furthermore, the diet of these patients is rich in glucose and fat, thereby increasing the level of adiponectin in serum (25). Our results revealed an increased level of leptin and a decreased level of adiponectin in the rats in the HFD group, but there was no significant difference among the treatment groups. The same trend can also be found in the plasma glucose and insulin concentrations, indicating that the lipid-related hormone level is moderately altered in treatment groups.

In most cases, obesity can trigger a series of inflammatory reactions. IFN-γ is a pro-inflammatory cytokine, and the reduction in IFN-γ levels improves metabolic outcomes in obesity (26). IL-10 is an anti-inflammatory cytokine that could prevent diet-induced obesity and suppress inflammatory responses (27). LPS is derived from gram-negative bacteria and can induce inflammatory responses in the host. An increase in LPS levels is directly associated with increased intestinal permeability (28). Our findings indicate a reduction in levels of LPS, IL-1β, and IFN-γ in groups administrated by pasteurized yogurt, coupled with an increase in IL-4 and IL-10 levels. This suggests that pasteurized yogurt has the potential to mitigate inflammation and contribute to the preservation of the gut barrier integrity in the host.

We performed 16S rDNA sequencing to determine the composition of gut microbiota in rats. The heat-killed BBMN68 and inulin could not significantly reverse the richness of the gut microbiota of HFD-fed rats. *LEfSe* analysis revealed that the SCFA-producing genera, *Roseburia* (29), *Romboutsia* (30), and *Eubacterium xylanophilum* (31) were enriched in the ND group. Furthermore, in the IN group, the genera enriched were negatively related to obesity like *Akkermansia* (32) and *Flavonifractor* (33) while in the MN68 + IN group, SCFA-producing genera *Intestinimonas* (34), *Oscillospira* (35) were also enriched. Overall, the combination of heat-killed BBMN68 and inulin increases the abundance of SCFA-producing bacteria in the gut microbiota, which increases the level of SCFAs in the body.

A study has shown that *Roseburia* cocultured with *Akkermansia* uses mucin to produce SCFAs (36), which enhances MUC2 expression in intestinal epithelial cells and increases mucus production (37). In addition, *Blautia* produces bacteriocins to inhibit pathogenic bacteria from colonizing the intestine (38). Our results revealed that beneficial bacteria like *Blautia* were enriched in the MN68 group. The proportion of SCFA-producing bacteria *Intestinimonas* and *Oscillospira* as well as negatively obese-associated genera *Christensenella* (39) were higher in the MN68 + IN groups compared to all HFD-induced groups. *Candidatus_Stoquefichus*, which is more abundant in THE MN68 + IN group, has shown to be negatively related to gut inflammation (40). These results suggested that the combination of heat-killed BBMN68 and inulin could modulate the gut microbiota and promote the growth of SCFA-producing bacteria.

Acetate and propionate can inhibit fat accumulation in adipose tissue via G protein-coupled receptor 43/free fatty acid receptor 2 (GPR43/FFAR2) (41). Cpt1 is a rate-limiting enzyme for the mitochondrial fatty acid β-oxidation. The dietary SCFA supplementation increases the expression of *Cpt1* in adipose tissue, which is associated with GPR43 signaling (42). A study has shown that the inactivated *Lactobacillus acidophilus* and mixed prebiotics increase the concentration of acetate and propionate in feces (43). Our results showed that the concentration of SCFA was significantly higher in the MN68 + IN group compared to the HFD group. Researchers have documented the anti-inflammatory properties of SCFAs, which encompass the suppression of pro-inflammatory cytokine synthesis and the mitigation of oxidative stress within the organism (44, 45). In congruence with these findings, our results reveal that groups exhibiting elevated SCFA levels, such as the MN68 + IN group, have decreased levels of pro-inflammatory cytokines (LPS, IL-1β, and IFN-γ) and heightened levels of anti-inflammatory cytokines (IL-4 and IL-10). These outcomes suggest that MN68 + IN treatment holds potential for ameliorating oxidative stress. Collectively, our findings indicate that heat-killed BBMN68 and inulin may augment SCFA synthesis and inhibit adipose tissue lipid deposition, thereby contributing to the prevention of obesity.

Recently, more advanced sequencing techniques have emerged. For example, ASV (Amplicon Sequence Variant) analysis offers improved resolution, error detection, and data utilization in identifying species (46) compared to OTU analysis. As a result, such method can be utilized in future studies to achieve more precise taxonomical analysis. A multi-omics integrated analysis can also be employed to identify specific pathways that are pivotal in the suppression of obesity.

## 5 Conclusion

The co-administration of heat-killed BBMN68 and inulin demonstrates efficacy in mitigating obesity development in Wistar rats. The treatment not only attenuates weight gain but also reduces the body fat rate and the size of adipocytes. Furthermore, the co-administration of heat-killed BBMN68 and inulin promotes the enrichment of SCFA-producing bacteria, such as the genera *Intestinimonas* and *Oscillospira*. The heightened abundance of these bacteria contributes to an increased concentration of SCFAs, mitigating inflammation and preventing weight gain. Our study provides an alternative approach to obesity prevention and highlights the potential of utilizing the combination of heat-killed BBMN68 and inulin as functional food ingredients, showing promise in improving obesity. Future studies can focus on exploring more physiological functions of heat-killed BBMN68 in different models or clinical cases.

## Data availability statement

The datasets presented in this study can be found in online repositories. The names of the repository/repositories and accession number(s) can be found at: https://www.ncbi.nlm.nih.gov/, PRJNA911911.

## Ethics statement

The animal study was approved by the Ethics Committee of Pony Testing Group Co., Ltd. The study was conducted in accordance with the local legislation and institutional requirements.

## Author contributions

SS: Conceptualization, Project administration, Writing – original draft. QZ: Conceptualization, Project administration, Writing – original draft. DL: Conceptualization, Project administration, Writing – original draft. HL: Methodology, Writing – original draft. HM: Methodology, Writing – original draft. XW: Project administration, Writing – original draft. YL: Supervision, Writing – original draft. PW: Supervision, Writing – original draft. RL: Supervision, Writing – original draft. HF: Project administration, Writing – original draft. YZ: Validation, Writing – original draft. YS: Validation, Writing – original draft. BF: Supervision, Writing – original draft. RW: Writing – review & editing.

## Footnotes

^1^http://rdp.cme.msu.edu/, version 2.13.
